# Vaccination against SARS-CoV-2 Does Not Protect against the Development of Anosmia in a Hamster Model

**DOI:** 10.3390/vaccines11101564

**Published:** 2023-10-05

**Authors:** Rachel A. Reyna, Jordyn Walker, Brooke Mitchell, Divya P. Shinde, Jessica A. Plante, Scott C. Weaver, Kenneth S. Plante

**Affiliations:** 1 Department of Pathology, University of Texas Medical Branch, Galveston, TX 77555, USA; 2 World Reference Center for Emerging Viruses and Arboviruses, University of Texas Medical Branch, Galveston, TX 77555, USAdimircha@utmb.edu (D.P.S.);; 3 Department of Microbiology and Immunology, University of Texas Medical Branch, Galveston, TX 77555, USA; 4 Institute for Human Infections and Immunity, University of Texas Medical Branch, Galveston, TX 77555, USA

**Keywords:** SARS-CoV-2, sequelae, anosmia, vaccination

## Abstract

Anosmia, a total or partial loss of the ability to smell, is one of the most frequently documented sequelae of severe acute respiratory syndrome coronavirus-2 (SARS-CoV-2) infection. Persistent anosmia is associated with a decrease in quality of life. Here, we assess the impact of virus lineage and vaccination status on anosmia development in the golden Syrian hamster model. To characterize anosmia driven by current variants, we assessed olfactory function in hamsters infected with SARS-CoV-2 lineages A, BA.2, BA.5, BQ.1, and BQ.1.1 using a buried food detection test. We found that significant anosmia occurs upon infection with all variants with a significant correlation between disease severity and degree of anosmia. Moreover, we found that vaccination with either the Pfizer (BNT16b2) or Moderna (mRNA-1273) mRNA vaccines does not protect against anosmia, despite protection against severe disease.

## 1. Introduction

Severe acute respiratory syndrome coronavirus-2 (SARS-CoV-2) is the etiological agent of the novel coronavirus 2019 disease (COVID-19). As of March 2023, over 676 million people have been infected with the virus, resulting in over 6.8 million associated deaths [1]. Anosmia is one of the most recognized symptoms of infection as it occurs in up to 85.6% of cases, regardless of disease severity [2,3,4,5,6,7,8,9,10]. This neurologic complication tends to be more common in women, adults, and outpatient cases [6,8,11]. While the majority of patients recover their sense of smell within 3–4 weeks, some may suffer persistent anosmia for more than a year following infection [11,12,13]. The effects of persistent anosmia are generating a societal hazard; COVID-19-associated anosmia and ageusia (loss of the sense of taste) are correlated with increased rates of depression and suicidal ideation [14]. The long-term complications of this neurologic sequela are yet to be fully understood.

The golden Syrian hamster is a well-characterized immunocompetent model for SARS-CoV-2 infection. Hamsters develop disease after infection with unmodified human clinical isolates of SARS-CoV-2, with low mortality and significant pathologic changes in the lung [15,16,17]. Moreover, these hamsters have been shown to develop significant transient anosmia similar to that seen clinically [18]. Previous studies have utilized this hamster model to assess the mechanisms driving anosmia [18,19,20]. Infection with SARS-CoV-2 induces severe damage to the olfactory epithelium within the nasal turbinates as early as 3 days post infection (dpi) [20]. This damage is transient, with significant epithelial regeneration by 21 dpi [20]. The severity of anosmia is correlated to the damage within the olfactory epithelium [18]. Infection within the olfactory epithelium likely occurs in the non-neuronal cells as olfactory sensory neurons do not express the angiotensin converting enzyme 2 (ACE2) receptor required for viral entry, resulting in deciliation and the associated anosmia [19,21,22,23,24].

The majority of anosmia studies have been conducted using the prototypical USA WA-1/2020 strain of SARS-CoV-2 as it is the best characterized isolate. However, the ancestral variant, USA WA-1/2020, lacks many of the adaptive changes associated with increased transmissibility, immune escape, and upper respiratory tract tropism that are present in more recent variants [25,26]. Understanding whether the contemporary circulating clinical variants are able to induce anosmia is crucial for fully understanding its driving mechanisms, as well as to act as a basis for the development and testing of medical countermeasures to alleviate its burden. Moreover, no work has assessed the efficacy of vaccination in reducing or preventing anosmia. In this study, we address both these gaps in knowledge using the golden Syrian hamster model and the buried food detection test.

## 2. Materials and Methods

### 2.1. Cells, Virus, and Vaccines

Vero TMPRSS2 cells (JCRB Cell Bank, Tokyo, Japan) were maintained using Dulbecco’s Modified Eagle Medium (DMEM) (Gibco, Waltham, MA, USA) supplemented with 10% fetal bovine serum (FBS) (R&D Systems, Minneapolis, MN, USA) and 1 mg/mL geneticin. SARS-CoV-2 (USA/WA-1/2020 [A], hCoV-19/USA/NY-MSHSPPV56475/2022 [BA.2], SARS-CoV-2/human/USA/COR-22-063113/2022 [BA.5], MDL-5125 [BQ.1], MDL-5115 [BQ.1.1]) variants were acquired from the World Reference Center for Emerging Viruses and Arboviruses (WRCEVA) at the University of Texas Medical Branch (UTMB) as aliquots of cell supernatant from infected Vero cells and were stored at −80 °C until use. Unused original Pfizer (BNT16b2) and Moderna (mRNA-1273) mRNA vaccines were acquired from clinics at UTMB through an institutional transfer and were stored at −80 °C until use.

### 2.2. Animal Experiments

Five to six-week-old male golden Syrian hamsters were purchased from Charles River. Hamsters receiving vaccines were intramuscularly vaccinated on study days −35 and −14 with 5 μg of either Pfizer (BNT16b2) or Moderna (mRNA-1273) mRNA vaccines diluted in 100 μL of sterile phosphate-buffered saline (PBS) or 100 μL of sterile Dulbecco’s Phosphate Buffered Saline (DPBS) (Sigma, St. Louis, MO) as an unvaccinated control. Vaccination doses were selected based on previous studies [27,28]. All hamsters were intranasally inoculated with 100 μL of either 10^4^ PFU of SARS-CoV-2 diluted in sterile PBS or PBS as a mock control. Cohorts were randomly selected for vaccination and infection groups. Hamsters were identified using metal ear tags and were fed standard laboratory chow over the course of the experiment. Body weights were measured using an Ohaus CX1201 portable scale. All hamsters were housed in the Animal Biosafety Level 2 (ABSL2) and ABSL3 facilities within the Galveston National Laboratory at UTMB. Nasal washes were performed just prior to euthanasia using a total of 200 μL of sterile DPBS. Euthanasia was conducted using a high flow rate of CO_2_ followed by thoracotomy. The right cranial lobe was collected from each hamster for viral titration. All animal studies are reviewed and approved by the Institutional Animal Care and Use Committee at UTMB and are conducted according to the National Institutes of Health guidelines.

### 2.3. Anosmia Testing

All hamsters were tested for anosmia using a buried food detection test, as previously described, on 5 dpi [18]. Briefly, an empty housing cage was prepared containing at least 3 cm of bedding. In one corner, a honey-flavored Teddy Graham (Nabisco) was buried about 1 cm below the surface of the bedding. A hamster was placed in the cage in the corner diagonal to the buried cookie, the lid closed, and a timer started. The time taken for the hamster to unbury and grasp the cookie was recorded. All hamsters were allowed a maximum of 5 min (300 s) to uncover the cookie. If the hamster was unable to detect the cookie by the end of the test, forceps were used to present the cookie directly to the hamster to determine whether the hamster recognized the cookie as food. Hamsters were excluded from the study if they did not demonstrate any investigative behavior. Each hamster was tested for anosmia only once to avoid learned behavior; euthanasia occurred just after testing.

### 2.4. Viral Titrations

Vero TMPRSS2 cells were seeded into 12-well plates and cultured overnight before infection. Hamster lung tissues were homogenized in DMEM supplemented with 2% FBS and then centrifuged for 5 min at 21,130× *g*. The tissues were diluted ten-fold serially in DPBS and 150uL of each dilution was placed in its associated well. The plates were incubated at 37 °C with 5% CO_2_ for one hour. After incubation, overlay consisting of 0.8% agarose LE (Promega, Madison, WI, USA) in 1x MEM (Gibco) supplemented with 4% FBS and 1% penicillin-streptomycin (Gibco) was added to each well. After 24 h (lineage A and mock infected) or 48 h (lineages BA.2, BA.5, BQ.1, and BQ.1.1) incubations, the plates were stained with 0.03% neutral red (Sigma-Aldrich, St. Louis, MO, USA) 4 hours prior to counting the plaques with the aid of a light box. Viral titers were calculated using the plaque count from the lowest dilution with clear, individual, and countable plaques and the following equation: PFU/mL = (plaque count)/((dilution factor)*(inoculum volume)). This concentration was converted to PFU/g by multiplying by the organ weight/mL. The log_10_ values of the viral titers were used for viral analysis.

### 2.5. Statistical Analysis

Statistical significance was calculated and graphs were generated using Prism version 9.5.1 (GraphPad, San Diego, CA, USA). A cutoff of *p* < 0.05 was deemed significant for all analyses. Weight change was analyzed using a two-way ANOVA followed by the Dunnett post hoc test. Anosmia was analyzed using a one-way ANOVA followed by the Dunnett post hoc test. Viral titers in nasal washes and lung tissue were log10 converted, then analyzed using a one-way ANOVA followed by the Tukey post hoc test. Samples below the limit of detection were considered to be half that value for statistical analysis and graphing purposes. Correlations between weight loss and anosmia and nasal wash and anosmia were determined using Pearson’s correlation.

## 3. Results

### 3.1. Omicron Variants Induce a Less Severe Disease in Golden Syrian Hamsters

**Figure 1:**
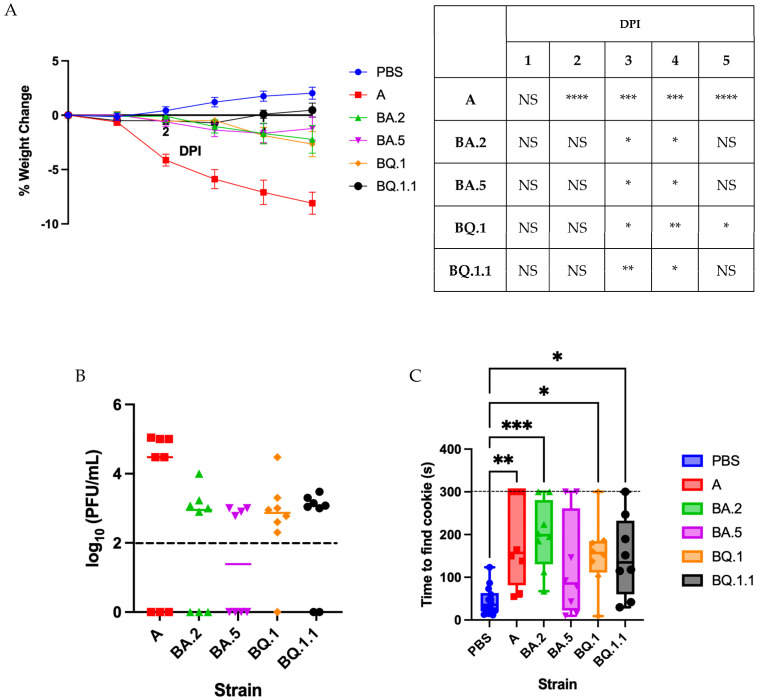
Omicron variants induce a milder acute disease than the ancestral variant in golden Syrian hamsters, yet still present with significant anosmia. (**A**) percentage weight change in hamsters intranasally infected with 10^4^ PFU of variant A, BA.2, BA.5, BQ.1, or BQ.1.1 SARS-CoV-2 or mock infected with PBS. *n* = 8 per infection group and *n* = 16 for PBS. Data presented as mean ± SEM. Significant differences in weight change as compared to the PBS group were determined using a two-way ANOVA followed by Dunnett’s multiple comparisons test; the degree of these significant differences is denoted in the table to the right of the graph. (**B**) viral load in nasal wash samples collected at 5 dpi. Symbols represent individual subjects, midlines represent the mean, and error bars represent the standard deviation. Dashed line represents the lower limit of detection for the assay. (**C**) the time taken for hamsters to find a buried cookie. Data presented as box plots with individual values. The midlines represent the median values and the error bars represent the range of maximum and minimum values. The maximum detection time was 300 s. *n* = 8 per infection group and *n* = 16 for PBS. Significance was analyzed using a one-way ANOVA followed by the Dunnett post hoc test. * *p* < 0.05, ** *p* < 0.01, *** *p* < 0.001, **** *p* < 0.0001. NS—not significant.

Male golden Syrian hamsters were intranasally inoculated with 10^4^ plaque forming units (PFU) of either lineage A, BA.2, BA.5, BQ.1, or BQ.1.1 SARS-CoV-2 or with PBS as a mock control (*n* = 8 per group). The A-infected hamsters presented with the most severe disease, with significant weight loss through 5 dpi (Figure 1A), while BQ.1.1-infected hamsters developed the mildest disease, with weight recovery beginning at 4 dpi. The BA.2-, BA.5-, and BQ.1-infected hamsters presented with a moderate disease phenotype, resulting in statistically significant yet minimal weight loss compared to the mock-infected control group. Nasal washes collected at 5 dpi (Figure 1B) demonstrated persistent viral titers in each infection group, with no significant differences between lineages.

### 3.2. Characterizing Anosmia with Recent Variants

Previous work has demonstrated that the lineage A USA WA-1/2020 produces significant anosmia in golden Syrian hamsters [18]. To determine whether the more recent Omicron variants also induce anosmia, hamsters underwent the buried food detection test on day 5 dpi, as previously described [18]. Significant anosmia was detected in all infection groups, as compared to mock-infected hamsters, except for BA.5-infected hamsters that strongly trended towards increased anosmia but failed to reach significance (Figure 1C). Hamsters that reached the 300 s maximum cutoff time were directly presented with the olfactory stimulant; all ignored the cookie, potentially indicating total anosmia. Using Pearson’s test, a significantly negative correlation was found between percentage weight loss and severity of anosmia (Table 1). There is also a mild, albeit non-significant, positive correlation between nasal wash titer and severity of anosmia.

### 3.3. Impact of Vaccination on Pathogenesis and Anosmia

**Figure 2:**
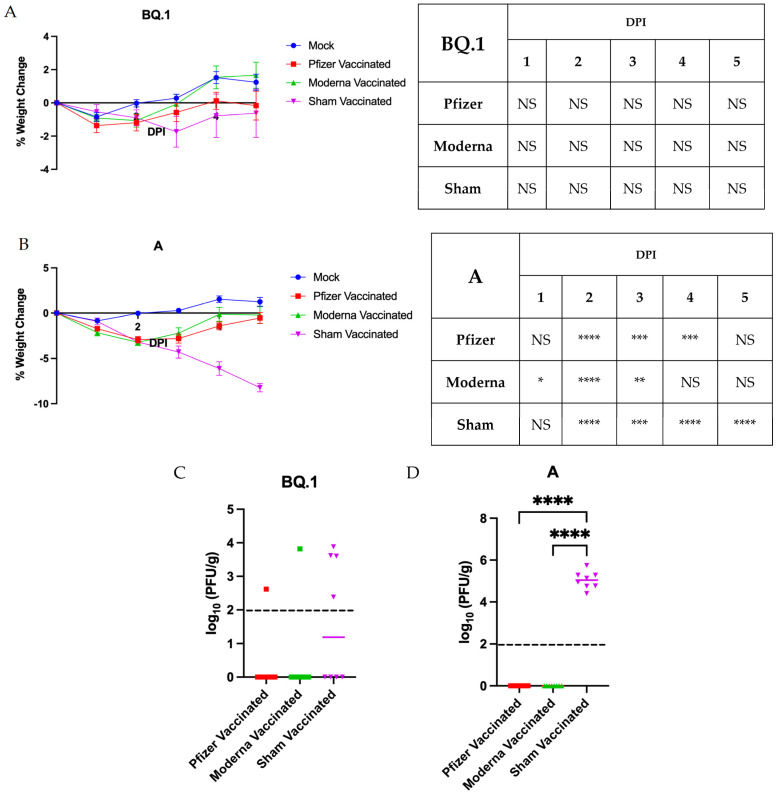
Vaccination with Pfizer (BNT16b2) or Moderna (mRNA-1273) prevents against severe disease in golden Syrian hamsters. (**A**,**B**) percentage weight change in vaccinated hamsters infected with 10^4^ PFU of BQ.1 or WA-1. *n* = 8 per infection group and *n* = 24 for mock. The mock group is pooled data from Pfizer, Moderna, and sham vaccinated uninfected controls. Data presented as mean ± SEM. Significance was determined using a two-way ANOVA followed by Dunnett’s multiple comparisons test; the degree of these significant differences is denoted in the table to the right of the graph. (**C**,**D**) lung titers collected at the endpoint (5 dpi). Significance was assessed using a one-way ANOVA followed by the Tukey post hoc test. * *p* < 0.05, ** *p* < 0.01, *** *p* < 0.001, **** *p* < 0.0001. NS—not significant.

To determine whether prior vaccination would reduce the severity of anosmia, male hamsters were intramuscularly vaccinated in a prime/boost strategy with either 5 μg Pfizer (BNT16b2) or Moderna (mRNA-1273) mRNA vaccines or with PBS as an unvaccinated control (*n* = 8 per group). This dose and schedule was selected as it is within the accepted range for hamsters and has been shown to induce significant seroconversion [27,28]. Two weeks after the boosting vaccination, hamsters were intranasally inoculated with 10^4^ PFU of either lineage A or BQ.1 SARS-CoV-2, or with PBS as a mock control. Data from hamsters receiving vaccination with either Pfizer, Moderna, or PBS that were then mock infected with PBS were pooled as the Mock group. The sham vaccinated group consists of pooled data from hamsters receiving a mock vaccination with PBS but infection with either lineage A or BQ.1 SARS-CoV-2. Both the Pfizer and Moderna vaccines conferred significant protection against weight loss following challenge with either lineage BQ.1 (Figure 2A) or lineage A (Figure 2B) SARS-CoV-2, compared to the sham vaccinated control groups. However, this protection was not complete; the vaccinated cohorts did lose a significant amount of weight compared to the sham vaccinated or mock challenged control group at one or more measured timepoints.

Lung tissues of BQ.1-infected hamsters collected at 5 dpi demonstrated no significant differences in viral titer between vaccinated and unvaccinated groups (Figure 2C). However, a significant difference in mean viral titer in the lung was noted for lineage A-infected hamsters; no virus was detected in the lungs of vaccinated hamsters, whereas high viral titers were present in sham vaccinated hamsters (Figure 2D). This is as expected; the efficacy of the BNT16b2 and mRNA-1273 vaccines has dropped with new variants [29,30].

**Figure 3:**
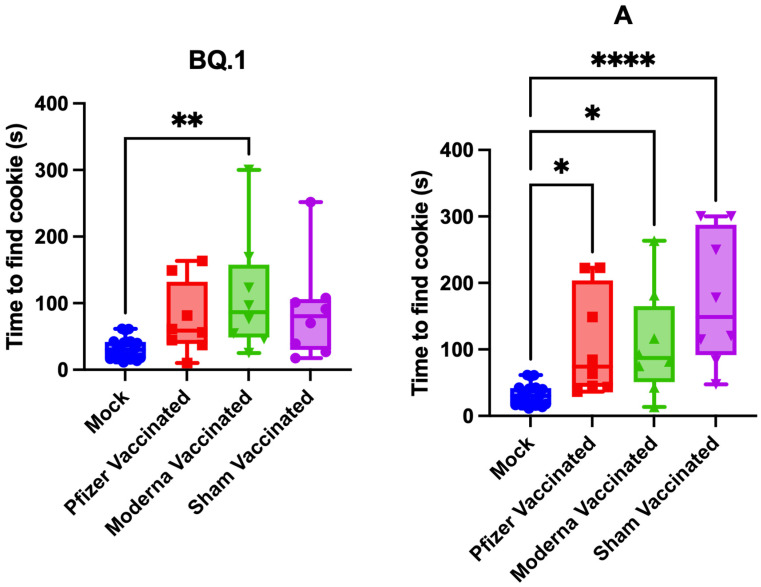
Vaccination does not significantly reduce severity of anosmia. The time taken for hamsters to find a buried Teddy Graham cookie at 5 dpi. Data presented as box plots with individual values. The maximum detection time was 300 s. *n* = 8 per infection group and *n* = 24 for the mock-infected group. Significance was analyzed using a one-way ANOVA followed by the Dunnett post hoc test. * *p* < 0.05, ** *p* < 0.01, **** *p* < 0.0001.

Anosmia testing was performed at 5 dpi (Figure 3). The BQ.1-infected hamsters did not develop significant anosmia, except for the Moderna vaccinated group, although a trend towards increased anosmia is noted. The WA-1-infected hamsters developed severe anosmia. Vaccination with either Pfizer or Moderna reduced, albeit not significantly, the severity of anosmia.

## 4. Discussion

Throughout the COVID-19 pandemic, anosmia has been a primary hallmark of SARS-CoV-2 infection. With an incidence of up to 85.6%, regardless of disease severity [2,3,4,5,6,7,8], SARS-CoV-2-mediated anosmia will have lasting effects on affected individuals due to its persistence and psychological impacts [11,12,13,14]. While progress has been made toward understanding the mechanism of anosmia [19,20,21,22,23], little to no work has been conducted to determine whether it can be prevented or mitigated. The objective of this study was to characterize anosmia resulting from infection with multiple Omicron variants as well as to determine whether vaccination with the original Pfizer (BNT16b2) or Moderna (mRNA-1273) mRNA vaccines can prevent or reduce the severity of the resulting anosmia.

Our study indicates that, consistent with clinical reports, the contemporary Omicron lineages (BA.2, BA.5, BQ.1, and BQ.1.1) cause a less severe disease than the ancestral lineage A (Figure 1) [31]. However, despite reduced weight loss and decreased viral load in nasal washes and lung tissue, lineages BA.2, BQ.1, and BQ.1.1 still induced significant anosmia at 5 dpi and lineage BA.5 trended towards the same. Moreover, a significant correlation was found between percentage weight loss and degree of anosmia (Table 1). While this is not necessarily clinically relevant as clinical infections with SARS-CoV-2 produce anosmia accompanying all levels of disease severity [2,3,4,5,6,7,8], this finding strengthens the golden Syrian hamster as a reliable model through which to study sequelae of infection.

Vaccination with either the Pfizer (BNT16b2) or Moderna (mRNA-1273) mRNA vaccines conferred partial protection against weight loss following challenge with either variant A or BQ.1 SARS-CoV-2 and reduced viral load in the lung following variant A challenge (Figure 2). However, vaccination did not prevent against anosmia (Figure 3). This work was conducted using the original Pfizer (BNT16b2) and Moderna (mRNA-1273) mRNA vaccines. These were selected as much of the American population received them during the early roll-out of vaccines. Additionally, the selected dosage schedule has been shown to induce significant seroconversion [27,28,32], confirmed in this study by protection against weight loss and viral clearance in the lung (Figure 2). However, this study was limited by inadequate sampling to determine neutralizing antibody concentrations. Future work should be conducted to reflect the current status of the population: people boosted with or double vaccinated with solely the bivalent versions of the vaccines, or people with immunity resulting from prior infection. As newer vaccines are designed, perhaps they may show increased effectiveness in reducing anosmia.

The inability of vaccination to prevent anosmia leads to the question as to whether vaccination would be able to prevent any of the hundreds of sequelae of infection. “Long COVID” is a broad term addressing the over 200 signs and symptoms affecting various organ systems throughout the body [33,34]. Over 65 million people are affected, with the majority being women or those developing only mild, acute disease [33,35]. Common sequelae include muscle weakness, fatigue, memory loss, cognitive impairments, brain fog, and depression [33,34,36]. Previous work indicates that hamsters may reflect these sequelae as well, with a histopathologic analysis of SARS-CoV-2-infected hamster brains demonstrating significant changes in the regions responsible for cognition, despite the absence of viral antigen [37]. These changes include persistent local and systemic inflammation as well as increased glial fibrillary acidic protein (GFAP) expression within the piriform cortex and hippocampus [37,38], which is a marker of astroglial activation following injury within the nervous system [39]. Moreover, a significant loss in neural spine density is noted within the hippocampus, leading to a loss of neural synapses [37]. Together, this indicates that hamsters may present with clinically relevant neurologic sequelae after SARS-CoV-2 infection. Further development of this model is crucial to not only understand these sequelae, but also to allow for efficacy studies for current vaccines and therapeutics in preventing their development or mitigating their impact.

As has been noted throughout the COVID-19 pandemic, sex plays a role in the severity of infection. While females tend to be more susceptible to long COVID, men are at higher risk for more severe acute disease and death [33,34,40]. The previous work characterizing anosmia in golden Syrian hamsters was conducted using females [18]. This study was performed with male hamsters and confirms this gender bias. In comparison to the previous study in females, the male hamsters we infected with the USA WA-1/2020 strain of SARS-CoV-2 developed more severe weight loss. Interestingly, their anosmia appeared to be slightly less severe than that of the females [18]. This strengthens the golden Syrian hamster as a clinically accurate model that can be reliably studied to understand the mechanisms driving disease.

In sum, despite their reduced severity of acute disease, the Omicron variants BA.2, BQ.1, and BQ.1.1 induce significant anosmia in golden Syrian hamsters. Moreover, vaccination with either the Pfizer (BNT16b2) or Moderna (mRNA-1273) mRNA vaccines is unable to prevent anosmia, despite reducing acute morbidity and, in some cases, viral load. Effort should be placed to use this reliable golden Syrian hamster model for the screening of therapeutics for their efficacy in reducing anosmia.

## Figures and Tables

**Table 1:** Pearson’s correlation values between anosmia and percentage weight loss or nasal wash titer. Pearson’s correlation as performed assessing the relation between the time taken to find the buried cookie and either percentage weight loss or log_10_ nasal wash titer.

	Percentage Weight Loss and Anosmia	Nasal Wash Titer and Anosmia
Pearson’s r	−0.389	0.235
*p* value	0.004	0.144

## Data Availability

Data are available upon request.
